# Iris and anterior chamber angle melanoma masquerading as benign melanocytic lesion

**DOI:** 10.1016/j.ajoc.2025.102353

**Published:** 2025-05-09

**Authors:** Mai-Linh N. Ton, Maria Del Valle Estopinal, Kapil Mishra

**Affiliations:** a University of California, Irvine School of Medicine, Irvine, CA, USA; b Department of Ophthalmology, Gavin Herbert Eye Institute, University of California, Irvine, CA, USA; c Departments of Pathology and Ophthalmology, Ophthalmic Pathology Division, University of California, Irvine, 101 The City Drive, South Orange, CA, 92868, USA

**Keywords:** Iris melanoma, Anterior chamber angle, Extrascleral extension, Genomic sequencing in uveal melanoma, Ocular melanocytic lesions, Immunohistochemistry, GNA11 mutation

## Abstract

**Purpose:**

Demonstrates the importance of integrating clinical findings, immunohistochemistry, and next-generation sequencing of somatic mutations in differentiating complex ocular melanocytic lesions. This study highlights distinct differences between benign conjunctival nevus, iris melanoma with extrascleral extension, and conjunctival melanoma with intraocular invasion.

**Observations:**

A 62-year-old male presented with painless vision loss and multiple pigmented lesions on the ocular surface. Initial impression was a benign conjunctival nevus but concerning for melanoma due to secondary changes of vision loss and increased intraocular pressure. Further investigation revealed an iris melanoma involving the anterior chamber angle with extrascleral extension. Enucleation confirmed atypical melanocytic cells infiltrating the iris stroma, anterior chamber angle, sclera, and conjunctiva. Immunohistochemistry showed SRY-Box Transcription Factor 10 (SOX10) and melanoma antigen (Melan-A) positivity. Gene sequencing identified a Guanine nucleotide-binding protein, alpha-11 (GNA11) mutation, suggesting uveal origin.

**Conclusions and importance:**

Highlights the value of a comprehensive diagnostic approach in evaluating ocular melanocytic lesions. The progression from an initial impression of benign conjunctival nevus to the discovery of iris melanoma with extrascleral extension emphasizes the need for thorough investigation, especially when pathological changes such as increased intraocular pressure and vision loss occur.

## 1 Introduction

Uveal melanoma, a rare but potentially deadly ocular cancer, presents with varying clinical outcomes depending on its anatomical origin and genetic profile. Iris melanomas, representing only 2–5 % of uveal melanomas,^1^ are generally more indolent with a 10-year metastasis rate of 6.9 %.^2^ In contrast, ciliary body and choroidal melanomas exhibit higher metastatic potential at 33.4 and 25.0 % respectively across a 10-year time period,^3^ and are associated with poorer prognosis. Genetic mutations play a crucial role in determining the course of the disease, with BReast CAncer gene 1 (BRCA1) associated protein 1 (BAP1) mutations linked to higher metastasis rates and worse outcomes. Eukaryotic Translation Initiation Factor 1A X-Linked (EIF1AX) and Splicing Factor 3b Subunit 1 (SF3B1) mutations are also associated with metastasis risk, but generally confer a better prognosis than BAP1 mutations.^4^ Risk factors for iris melanoma include fair skin, light iris color, inability to tan, and northern European ancestry.^5^, ^6^, ^7^ While most iris melanomas are asymptomatic, advanced cases may present with secondary glaucoma.^8^^,^^9^ Once metastasis occurs, regardless of the primary tumor location, the prognosis is poor, with a 1-year overall survival rate of uveal melanomas of about 50 % for metastatic cases.^10^ Compared to other forms of uveal melanoma, iris melanomas generally have a more favorable outlook, with mortality rates 5–10 times lower than those of ciliary body and choroidal melanomas.

Uveal melanomas generally exhibit either spindle cell or epithelioid morphology, with varying degrees of pigmentation, and can be categorized into iris, ciliary body, or choroid subtypes, each with distinct metastatic potential. Depending on the lesion, the cells can be amelanotic, lightly pigmented, or heavily pigmented. Blue nevi are characterized by dendritic melanocytes in the deeper dermis or sclera. Conjunctival melanomas represent 5 % of all ocular melanoma, arising in the majority of cases from conjunctival melanocytic intraepithelial lesions (primary acquired melanosis (PAM) with atypia). Given the complexity of the eye's structure and the potential for tumor metastasis or extension into adjacent tissues, a thorough combination of histological and molecular characterization is essential to accurately identify the tumor's cell of origin, assess its molecular profile, and guide predictions about its metastatic behavior and response to surgical or medical interventions. Histologic examination is essential to differentiate high-grade melanomas of the choroid and ciliary body from the more indolent iris melanomas, which have lower metastasis rates. Here, we present a case of a 62-year-old male with painless vision loss and multiple ocular surface pigmented lesions with initial impression of a benign conjunctival nevus. This lesion shared features of both conjunctival melanoma and uveal melanoma with extrascleral extension, and panel testing was performed for commonly found mutations in uveal melanoma.

## 2 Case presentation

A 62-year-old male was referred to our clinic with painless vision loss in the right eye over the last year in the setting of multiple ocular surface pigmented lesions. His past medical history included diabetes mellitus without retinopathy. He reported he could see pigmented lesions on his eye for about 2–3 years. His best-corrected visual acuity was light-perception only in the right eye and 20/20 in the left eye. Intraocular pressures were 25 mmHg in the right eye and 12 mmHg in the left eye.

**Fig. 1:**
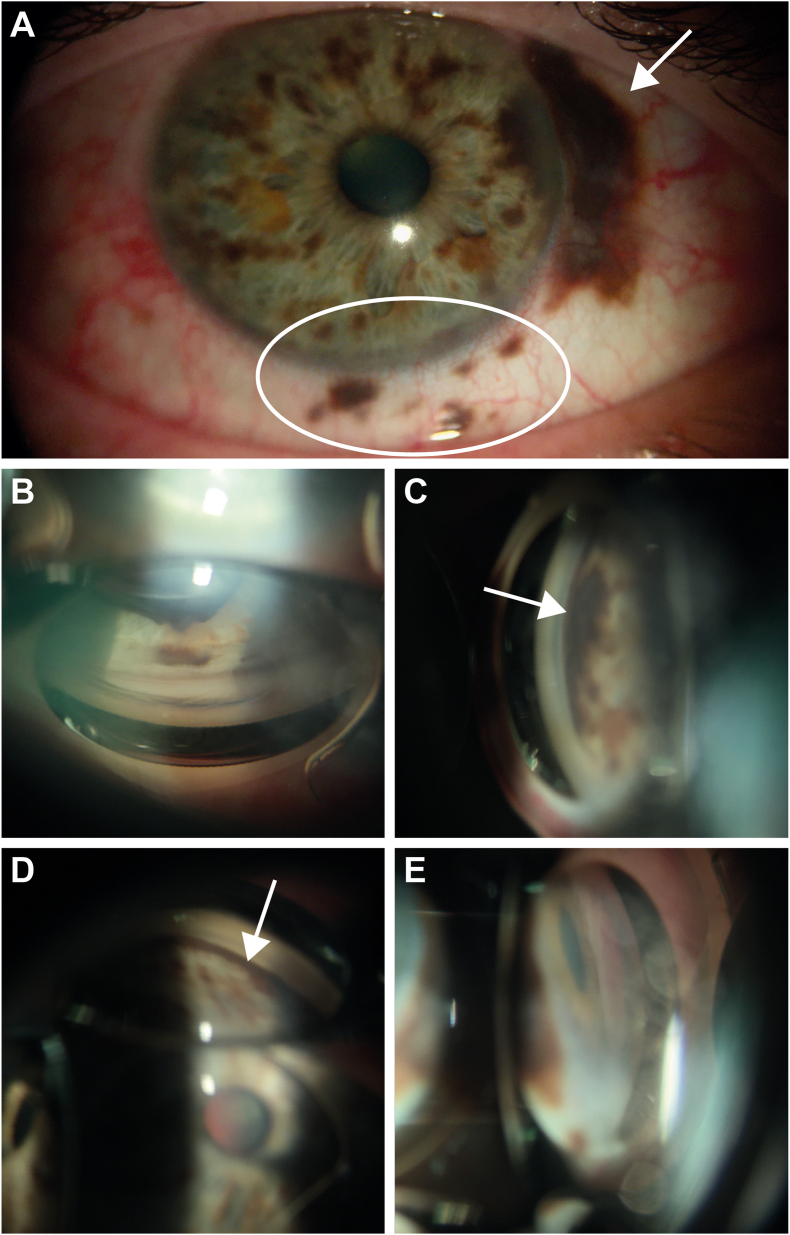
**Slit-lamp photos of pigmented iris and extrascleral mass. A**) Right eye slit-lamp photo highlighting the multifocal pigmented conjunctival perilimbal lesions. The arrow points to the larger extrascleral extension, and the white circle surrounds several extrascleral satellite lesions. Gonioscopic view of the right eye showing the **B**) superior quadrant without angle involvement, **C**) nasal quadrant with significant angle involvement (denoted by white arrow), **D**) inferior quadrant with significant involvement (denoted by white arrow), and **E**) temporal quadrant without significant angle involvement.

Slit lamp examination of the right eye revealed multifocal pigmented conjunctival versus subconjunctival perilimbal lesions nasally (Fig. 1A). There was no heterochromia. The unaffected left eye was normal with only a few iris freckles. In the right eye, there was a subconjunctival elevated melanotic lesion from 2 to 4 o'clock at the limbus, satellite focal lesions at 2 o'clock and several limbal lesions from 4 to 6:30 o'clock, as well as possible engorged sentinel vessels nasally. The iris of the right eye had dark brown pigmented flat lesions temporally, inferiorly, and nasally involving the angle structures (Fig. 1B–E). Fundus examination revealed an excavated disc of the right eye consistent with severe glaucoma. Examination of the left eye was unremarkable. B-scan ultrasound and ultrasound biomicroscopy did not demonstrate a ciliary body or choroidal lesion.

The patient underwent a conjunctival and scleral biopsy of one of the perilimbal lesions. At the time of biopsy the lesions were noted to be subconjunctival. A region of superonasal conjunctiva taken overlying the superior-most superonasal satellite lesion at 2 o'clock was taken as well as 2 mm segment of the sclera surrounding the pigmented scleral lesions. Initial histopathologic assessment suggested either a benign scleral lesion such as blue nevus or a bland iris melanoma. An additional subconjunctival lesion biopsy and iris fine-needle aspiration biopsy was obtained from a separate pigmented nasal perilimbal lesion at the nasal iris thickening. Examination revealed bland spindle-shaped melanocytic cells were positive for SRY-Box Transcription Factor 10 (SOX10) immunohistochemical (IHC) study, mixed with melanophages (positive for cluster of differentiation 68 (CD68)). Preferentially expressed antigen in melanoma (PRAME) IHC study was negative. Conjunctival intraepithelial dendritic melanocytic proliferation without high-grade atypia was also noted. The overall histologic findings were compatible with a proliferation of atypical predominantly spindle-shaped melanocytes involving conjunctival stroma and sclera.

Due to the concern for a uveal malignancy with extraocular extension causing near complete vision loss, and severe glaucoma from angle involvement, enucleation was recommended and the patient elected to proceed. The enucleation was performed while preserving the inferonasal and inferior cuff of conjunctiva with pigmentation on the globe. A 360-degree limbal peritomy was created elsewhere, and the extraocular muscles were detached, with the rectus and inferior oblique muscles secured for later reattachment. After removing the globe, a silicone sphere wrapped in donor sclera was inserted into the orbit, and the muscles were reattached. The procedure concluded with layered closure of Tenon's capsule and conjunctiva, followed by placement of a conformer and tarsorrhaphy. The enucleated eye was sent to combined IHC studies as well as panel gene sequencing to detect somatic mutations to determine if the lesion was of uveal origin. The margins were not involved by melanocytic cells.

**Fig. 2:**
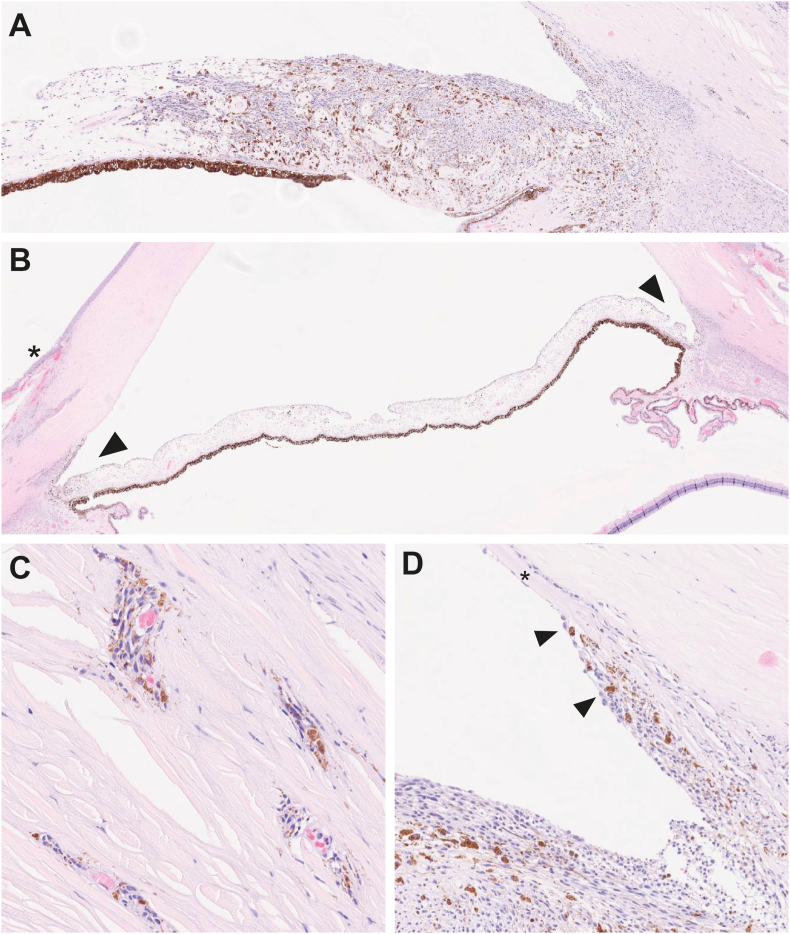
**Histological examination of the enucleated eye, demonstrating involvement of anterior chamber angle structures. A)** A 2.60x magnification hematoxylin and eosin (H&E) image, showcasing a predominantly plaque-like lesion that is slightly elevated. **B**) A 1.16x H&E-stained low-power view of the inferior calotte of the eye globe, with involvement of anterior chamber angle structures at both temporal and nasal quadrants (arrows). The asterisk marks the site of a previous biopsy. **C**) A high-resolution H&E-stained image at 19.83x magnification, revealing atypical spindle cells located in the perivascular spaces within the anterior emissary canals. **D**) H&E image at 8.81X magnification showing peripheral Descemet membrane (asterisk) revealing melanoma cells obscuring anterior chamber angle structures and lining Schwalbe's line (arrow).

Histologically, a 3.5 mm (basal) x 0.512 mm (thickness) plaque-like proliferation of cohesive atypical spindled melanocytic cells was infiltrating the iris stroma, with distortion and focal effacement of its architecture (Fig. 2A). Morphologically, the atypical cells were characterized by elongated nucleus with longitudinal groove, and eosinophilic cytoplasm (Spindle A cells). Rare neoplastic cells demonstrated central nucleolus (Spindle B cells). There was no definite evidence of atypical mitoses or necrosis. Involvement of the anterior chamber angle structures by neoplastic cells with secondary extension to the adjacent intrascleral canals and bulbar conjunctiva was observed at both temporal and nasal quadrants (Fig. 2B). The neoplastic cells were surrounding small vascular channels and infiltrating conjunctival stroma (Fig. 2C) and the anterior chamber angle structures, with atypical melanocytic cells lining the Schwalbe line (arrow) (Fig. 2D). The extensive involvement of anterior chamber angle structures by atypical melanocytic cells supports the clinical history of severe right-eye glaucoma. In addition, the optic nerve revealed atrophic changes suggestive of glaucomatous neuropathy. The optic nerve was not involved by neoplastic cells.

**Fig. 3:**
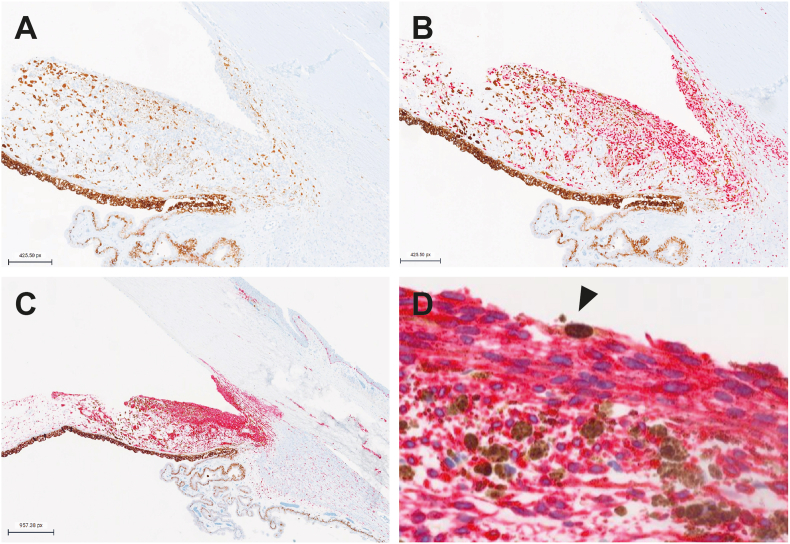
**Immunohistochemical (IHC) analysis of iris tumor cells. A**) Preferentially expressed antigen in melanoma (PRAME) IHC study showing negative staining in tumor cells at 5.88x magnification. **B)** SRY-Box Transcription Factor 10 (SOX10) IHC study highlighting melanoma cells in the stroma of the iris at 5.88x magnification. **C)** melanoma antigen (Melan-A) IHC study illustrating the iris tumor cells and their infiltrative component through the angle structures, sclera, and limbus at 2.61x magnification. **D)** Combined Melan-A Red (red)/Ki-67 (brown) IHC study demonstrating a low proliferation index within tumor cells, with rare Melan-A-positive cells showing positive proliferation (arrow) at 44.62x magnification.

The combined clinical and histologic findings raised suspicions for an iris ring melanoma with extrascleral extension and prompted deep gene profiling to differentiate between benign nevus and iris ring melanoma. Immunohistochemically, PRAME expression by immunohistochemistry was negative in the melanocytic cells (Fig. 3A), which was supported by the negative results of Decision-Dx-PRAME reverse transcription polymerase chain reaction. SOX10 (Fig. 3B) and melanoma antigen (Melan-A) (Fig. 3C–D) highlighted neoplastic cells infiltrating, circumferentially, the trabecular meshwork, anterior chamber, and focally the ciliary body. Foci of residual tumor cells in two quadrants of the stroma of bulbar conjunctiva were also observed. The proliferation index, Ki-67 was positive in rare, atypical cells (Fig. 3D). Furthermore, neoplastic cells were B-Raf Proto-Oncogene (BRAF) V600E negative. Sixty to 70 % of the lesional cells were weakly nuclear BAP1-positive. Some CD68-positive melanophages were intermixed with the atypical melanocytes.

Final gene sequencing of the tumor using DecisionDx-UMSeq panel testing for common uveal melanoma mutations in BAP1, cysteinyl leukotriene receptor 2 (CYSLTR2), EIF1AX, GNA11, Guanine Nucleotide-binding Protein, Q polypeptide (GNAQ), phospholipase C beta 4 (PLCB4), and SF3B1^11^ revealed a Class 1A melanoma with 626A > T missense mutation in GNA11. GNA11 mutations occur in 40–45 % in uveal melanoma and is involved in constitutive activation of signalling pathways downstream of G-protein-coupled receptors.^12^ The patient underwent magnetic resonance imaging with and without contrast, as well as computed tomography scans of the chest and abdomen which showed no signs of metastasis. For a Class 1A designation, the patient is undergoing yearly liver surveillance for 5 years to monitor for signs of metastasis.

## 3 Discussion

Iris melanomas are known to often be spindle-shaped and bland which could mimic more benign lesions of the eye. In this case, atypical spindle cells were present in the perivascular spaces within the anterior emissary canals, indicating the tumor's ability to penetrate through to the sclera. This extraocular extension, though rare in iris melanomas, demonstrates the tumor's aggressive nature and its capacity to invade adjacent ocular structures. In this case, the clinical findings of glaucoma secondary to extensive involvement of the anterior chamber angle structures, along with the pathological evidence of painless vision loss, indicate a more malignant lesion than what was initially suspected. Extensive pathological testing indicated a low-grade spindle cell melanoma with rare extraocular extension. Extraocular extension is a feature observed in only 3 % of iris melanoma cases.^9^ Advanced diagnostic tools, including DecisionDx-UMSeq, help determine lesions with low or high metastatic potential. These diagnostic tools can also accurately identify genetic mutations associated with specific melanomas such as uveal melanoma, and prognostic characteristics of the tumor. GNAQ, Guanine nucleotide-binding protein subunit alpha-11 (GNA11), EIF1AX, and BAP1 are commonly identified mutations in iris melanoma.^13^ In this case we detected no mutations in BAP1. BAP1 mutations are reported in 47 % of uveal melanomas and linked to early metastasis and poorer prognosis due to increased epithelial to mesenchymal transition^14^^,^^15^.

Tumor extraocular extension of uveal melanoma can mimic benign melanocytic lesions of the sclera and conjunctiva including nevocellular nevus, ocular melanocytosis and blue nevus, among others. Also, a large extraocular extension from uveal melanoma can look clinically similar to conjunctival melanoma with intraocular invasion. Typically in uveal melanoma, extrascleral extensions would advance into the subconjunctival or stromal space and lack conjunctival feeder vessels. Iris melanoma typically shows a plaque-like proliferation of cohesive atypical spindled melanocytic cells infiltrating the iris stroma. Conjunctival melanomas are characterized by atypical melanocytes forming nests along the basal layer of the conjunctival epithelium, with potential invasion into subepithelial layers. Benign conjunctival nevi usually demonstrate well-circumscribed nests of bland melanocytes without significant atypia. Ocular melanocytosis shows increased melanocytes in ocular tissues without forming discrete lesions.

Importantly, iris nevus and iris melanoma can share the same genetic abnormalities, so a comprehensive approach is necessary to differentiate malignant from benign lesions. Iris melanomas are usually low-grade tumors with usually bland architecture and histopathologic features similar to those observed in iris nevus. Histological differentiation of ocular melanocytic lesions is crucial for accurate diagnosis and appropriate management. Given the complexity of the eye's structure and the potential for tumor metastasis or extension into adjacent tissues, a thorough combination of histological and molecular characterization is essential to accurately identify the tumor's cell of origin, assess its molecular profile, and guide predictions about its metastatic behavior and response to surgical or medical interventions.

## CRediT authorship contribution statement

**Mai-Linh N. Ton:** Writing – review & editing, Writing – original draft, Visualization, Data curation. **Maria Del Valle Estopinal:** Writing – review & editing, Supervision, Methodology, Investigation, Data curation. **Kapil Mishra:** Writing – review & editing, Supervision, Project administration, Methodology, Investigation.

## Patient consent

Written consent to publish this case has not been obtained. This report does not contain any personal identifying information.

## Acknowledgements and disclosures

The authors acknowledge support to the Gavin Herbert Eye Institute at the University of California, Irvine from an unrestricted grant from Research to prevent blindness and the Charles D. and Judith K. Fritch Endowment.

## Authorship

All authors attest that they meet the current ICMJE criteria for Authorship.

## Declaration of competing interest

The authors declare that they have no known competing financial interests or personal relationships that could have appeared to influence the work reported in this paper.
